# Formulation, Characterization, and Antioxidant Properties of Chitosan Nanoparticles Containing Phenolic Compounds from Olive Pomace

**DOI:** 10.3390/antiox13121522

**Published:** 2024-12-12

**Authors:** Ilaria Fierri, Roberto Chignola, Chiara Stranieri, Edoardo Giuseppe Di Leo, Maria Bellumori, Sara Roncoletta, Alessandro Romeo, Federico Benetti, Anna Maria Fratta Pasini, Gianni Zoccatelli

**Affiliations:** 1 Department of Biotechnology, University of Verona, 37134 Verona, Italy; ilaria.fierri@univr.it (I.F.); roberto.chignola@univr.it (R.C.); sara.roncoletta@univr.it (S.R.); 2 Department of Medicine, Section of Internal Medicine D, University of Verona, 37134 Verona, Italy; chiara.stranieri@univr.it (C.S.); edoardogiuseppe.dileo@univr.it (E.G.D.L.); annamaria.frattapasini@univr.it (A.M.F.P.); 3 Department of NEUROFARBA, University of Florence, 50019 Sesto Fiorentino, FI, Italy; maria.bellumori@unifi.it; 4 Department of Computer Science, University of Verona, 37134 Verona, Italy; alessandro.romeo@univr.it; 5 ECSIN-ECAMRICERT SRL Laboratory, 35127 Padua, Italy; f.benetti@ecamricert.com

**Keywords:** antioxidants, nanoencapsulation, hydroxytyrosol, ionotropic gelation, lyophilization, nanoparticles, oxidative stress

## Abstract

Olive phenolic compounds like hydroxytyrosol (OH-Tyr), tyrosol (Tyr), and their precursors have different health-promoting properties, mainly based on their strong antioxidant capacity. However, their presence in extra-virgin olive oil (EVOO) is scarce since they are primarily contained in the by-products of oil production, such as olive pomace (OP). The aim of this work was to extract and encapsulate OP phenolic compounds into chitosan–tripolyphosphate nanoparticles (NPs) using an ionotropic gelation lyophilization approach to increase their resistance to environmental and chemical stress. NPs resulted in a monodisperse (PDI: 0.21) population of cationic NPs (ζ-potential: 33 mV, size: 229 nm) with an encapsulation efficiency (EE%), expressed as total phenolic content (TPC) and total OH-Tyr + Tyr content, of 64–65%. Mannitol and maltodextrin DE 19 (MD-19) were evaluated as lyoprotectants to counteract irreversible NP aggregation during lyophilization. The NP powder freeze dried using 0.7% of MD-19 showed the best performance, returning a monodispersed population of particles after rehydration. The antioxidant capacity of the obtained NPs was confirmed both in cell-free assays and in a THP-1 cell model of oxidative stress. This method represents a promising way to deliver health-promoting olive phenols for nutraceutical purposes and, hence, to valorize otherwise wasted by-products.

## 1. Introduction

According to the 2019 summary report on food loss and waste reduction presented by the Food and Agricultural Organization (FAO), 13.8% of global food is wasted throughout the food chain, with the most significant losses registered for retail, food service, and households [1]. In this scenario, food waste valorization is considered a noble approach, as many food by-products are rich in natural antioxidants or functional food ingredients that can still be extracted and further processed [2,3]. Olive pomace (OP), which is a semisolid and polluting biomass composed of olive stones, peels, and pulp obtained from olive oil processing, is particularly promising considering the amount of waste produced compared to the final yield of virgin olive oil collected at the end of the milling process (i.e., 12–20% of fruit weight) [4]. Extra-virgin olive oil (EVOO) is considered one of the main promotors of the health benefits ascribed to the Mediterranean diet, especially in preventing cardiovascular diseases and metabolic disorders, thanks to its elevated concentration of monounsaturated fatty acids (MUFAs) [5]. Part of the beneficial capacity of EVOO appears to be similarly related to olive secondary metabolites, e.g., hydroxytyrosol (OH-Tyr), tyrosol (Tyr), and their secoiridoids [6], which the European Food Safety Authority (EFSA) has declared capable of contributing to the protection of blood lipids from oxidative stress. More specifically, it is clarified that “the claim may be used only for olive oil which contains at least 5 mg of hydroxytyrosol and its derivatives (e.g., oleuropein complex and tyrosol) per 20 g of olive oil” [7]. Nevertheless, no more than 0.5% of the phenols contained in the intact fruit are transferred to EVOO, while the most significant part is lost during the process and can be found in its by-products [8]. Despite their potential, these molecules are easily prone to degradation, in particular when subjected to heat and when dissolved in ionic aqueous solutions [9], hindering their commercial use.

Encapsulation is an extensively used approach employed to defend liable molecules from environmental stressors, to increase shelf-life, and to ameliorate the functional properties of target compounds [10]. In particular, encapsulation has been extensively described as a strategy to stabilize extracts rich in phenolic compounds obtained from the valorization of food wastes and by-products [11]. In addition, encapsulation is also widely used to provide additional benefits, i.e., improving bioavailability, masking unpleasant flavors and tastes, and achieving controlled release in the gastrointestinal tract [11]. However, there is little information about OP polyphenol encapsulation attempts in the literature [12,13,14], and, to the best of our knowledge, no records exist regarding OP nanoencapsulation using chitosan (CS) as a wall material. CS is a natural cationic polysaccharide deriving from the deacetylation of chitin, the main component of the exoskeleton of several arthropods and the cell walls of some filamentous fungi. More specifically, this linear biopolymer is particularly used as an encapsulating material in both foods and pharmaceuticals thanks to its stability, its wound-healing properties [15], and its capacity to form ionic and covalent bonds with salts, proteins, and other polysaccharides [16]. The ionotropic gelation method, which is based on the interaction between a cationic polysaccharide like CS and several different counterions, such as pentasodium tripolyphosphate (NaTPP), bisulfite, or citrate salts, is a cheap, green, and mild approach to encapsulate polyphenols into nanosized particles with limited energy costs and without the need to use toxic solvents. Despite these advantages, its application is hindered by the nanoparticles’ (NPs’) tendency to agglomerate [16], which greatly limits their resuspension once freeze dried [17,18]. To the best of our knowledge, powder CS/NaTPP formulations containing OP polyphenols have never been reported, nor have attempts to employ maltodextrin as a lyoprotectant to help preserve CS NPs been carried out.

In this work, we aimed to encapsulate an ethanolic OP extract into CS and NaTPP NPs and transform the colloidal formulation into a powder. The effects of the extract on the NP characteristics in liquid form, as well as the lyoprotective capacity of maltodextrin and mannitol, were investigated to optimize the process conditions. The biological activity of the dried NPs was finally proven in terms of antioxidant capacity in both a cell-free environment and in the THP-1 cell line model of oxidative stress.

## 2. Materials and Methods

### 2.1. Materials

Pitted olive pomace (OP) from several cultivars (Leccino, Casaliva, Grignano, Favarol, Pendolino) was collected from the POG—Produttori Agricoli Gardesani—olive mill (Caprino Veronese, Verona, Italy) during the 2022 crop season. The milling process was carried out using a two-phase decanter (Leopard Series, Pieralisi Group S.p.A., Jesi, Italy). Low-molecular-weight chitosan (CS, deacetylation degree 93%) was purchased from Giusto Faravelli S.p.A. (Milan, Italy). Maltodextrin DE (dextrose equivalent)-19 (MD-19) was provided by Agrana AG (Vienna, Austria). Pentasodium tripolyphosphate NaTPP, Folin–Ciocalteu reagent, ABTS, TPTZ, ter-butyl hydroperoxide (TBHP), ammonium iron (II) sulfate hexahydrate, formic acid, sulfuric acid, acetic acid, 3-hydroxytirosol (OH-Tyr), tyrosol (Tyr), gallic acid (GA), ascorbic acid (AA), dihydrorhodaminine 123, mannitol, acetonitrile, formic acid, methanol, and ethanol were purchased from Merck KGaA (Darmstadt, Germany). The PE Annexin V Apoptosis Detection Kit I for the determination of cell viability and 7-amino-actinomycin D were purchased from BD Biosciences (BD Pharmingen, Franklin Lakes, NJ, USA). A CellROX Deep Flow Cytometry Assay Kit was purchased from Life Technologies (Grand Island, NY, USA).

### 2.2. OP Characterization

#### 2.2.1. OP Lyophilization, Moisture Content, and Water Activity

Fresh batches of OP were frozen and lyophilized within 24 h of collection using a Pascal Lio5P (Milan, Italy) freeze-drying device at a temperature of −50 °C and a pressure of 0.050 mbar for 5 days. The moisture content of fresh OP was assessed gravimetrically using an M60-VN natural convection stove (Memmert, Germany) at 105 °C for 24 h. The water activity (aw) of lyophilized OP (LOP) was measured soon after removal from the freeze-drying chamber using a HygroPalm HP2-Aw-A measuring device coupled with an HC2-AW measuring station (Rotronic, Bassersdorf, Zurich, Switzerland). LOP samples were finely powdered using a kitchen grinder (Braun Multiquick, Neu-Isenburg, Germany) and stored in the dark in vacuum bags.

#### 2.2.2. LOP Extraction and Hydrolysis

LOP extraction was conducted following the protocol suggested by Bellumori et al. [19]. Briefly, 40 g of LOP was extracted twice using a total volume of 1.6 L of EtOH:H_2_O (8:2), keeping the mixture under steady rotation for a total of 2 h. The supernatant was collected after centrifuging for 10 min at 4.400 g and 4 °C, and the pellet was subjected to a second extraction. The OP fluid extract (OPFE) was subsequently concentrated by means of a Buchi R-200 rotatory evaporator (Buchi Corporation, Flawil, Sankt Gallen, Switzerland) and brought to a final volume of 200 mL and a final EtOH concentration of 30% *v/v*. The OPFE was stored at −20 °C until use. Only for the quantification of total OH-Tyr and Tyr content by HPLC-DAD (see below), OPFE was subjected to acid hydrolysis, according to Bellumori and coworkers [20]. First, 200 μL OPFE was hydrolyzed with 400 μL of 1 M H_2_SO_4_ solution at 80 °C for 2 h. Then, 400 μL of distilled water was added to stop the hydrolysis before HPLC-DAD analysis.

#### 2.2.3. Total Polyphenol Content (TPC)

The total phenolic content of OPFE was measured through a Folin–Ciocalteu assay on 96-well microtiter plates as described by Zanoni and coworkers [21]. Briefly, 5 μL of sample or standard was mixed with 150 μL of diluted Folin–Ciocalteu reagent (1:10) and 40 μL of sodium carbonate. The microplate was incubated for 30 min in the dark at 37 °C, and the absorbance was measured using a Tecan Infinite PRO 200 plate reader (Tecan Trading AG, Switzerland) at a 750 nm wavelength. All results were expressed as milligrams of gallic acid (GA) equivalents per g of LOP dry weight (dw) (mg GAE/g OP dw) or, for NP liquid formulations, as milligrams of gallic acid (GA) equivalents per milliliter (mg GAE/mL), using a gallic acid calibration curve from 1.0 to 0.015 mg/mL. All experiments were performed in triplicate.

#### 2.2.4. OP HPLC-DAD Analysis

OH-Tyr and Tyr quantification was performed using an HPLC Jasco Extrema LC-4000 (Jasco Europe, Cremella, Italy) instrument equipped with a 4010 Diode Array Detector and a Poroshell 120 EC C18 150 × 3 mm i.d., 2.7 μm particle size (Agilent Technologies, Palo Alto, CA, USA) column. Gradient elution was conducted using a flow rate of 0.400 mL/min for 65 min at 25 °C, using H_2_O acidified with formic acid (pH = 3.22—solvent A) and acetonitrile (solvent B). The gradient was as follows: 5–40% B (0–45 min), 40–100% B (45–50 min), 100% B (50–53 min). Peak areas were plotted against corresponding concentrations (R2 = 0.999) of a Tyr six-point calibration curve. For the quantification of OH-Tyr, the regression results were corrected by multiplying the Tyr concentrations by 0.65, as indicated elsewhere [20]. Data acquisition was performed using a Jasco ChromNAV v.2.03.06.

### 2.3. Nanoparticle Formation and Characterization

#### 2.3.1. Solution Preparation

Chitosan was solubilized at 0.5% *w/v* concentration in a diluted acetic acid solution (1% *v/v*), while NaTPP was solubilized at 0.2% *w/v* in double-deionized water. Both solutions were kept under continuous stirring overnight to permit the complete hydration of the powders and stored at 4 °C until use.

#### 2.3.2. Ionotropic Gelation

NPs were prepared through ionotropic gelation, adapting a protocol described by Soltanzadeh and coworkers [22]. Different concentrations of OPFE were gently stirred in the CS solution for 1h in the dark. Subsequently, cold TPP was added to the solution at a flow rate of 5 mL/min by means of a peristaltic Econo Pump EP-1 (Bio-Rad, Hercules, CA, USA) equipped with a 3.2 mm ID capillary (ratio CS: OPFE: TPP 9:1:10). After 2 min of gelation, the solution was transferred in tubes and subjected to a total of 3 cycles of sonication for 60 s each, alternating with 60 s of rest in ice, at a 60% amplitude using a Microson Ultrasonic Liquid Processor XL-2000 (Misonix Inc., Farmingdale, NY, USA) equipped with a P-1-01 probe. Liquid NPs (liqNPs) were ultimately kept in ice for 10 min before being stored at 4 °C.

#### 2.3.3. Dynamic Light Scattering (DLS) Analysis

The Z-average, ζ-potential, and polydispersity index (PDI) of NPs were measured using a Malvern Zetasizer Nano-ZS (Malvern Instruments, Worcestershire, UK) at 25 °C. Small aliquots of fresh formulation were diluted prior to the investigation with double-deionized water. Each DLS analysis was performed in triplicate using automatic settings and disposable polystyrene cuvettes. ζ-potential evaluations were performed at ambient temperature using the Smoluchowsky approximation.

#### 2.3.4. Nanoparticle Tracking Analysis (NTA)

The average dimensions of NPs were also measured by NTA using a NanoSight NS300 (Malvern, Worcestershire, UK). All samples were diluted in double distilled water and subjected to 3 cycles of 60 s, each recorded at 25 FPS and 20 μL/min flow at ambient temperature. Data analysis was carried out using the house software NTA 3.4 Build 3.4.003.

#### 2.3.5. Atomic Force Microscopy (AFM)

AFM analysis was performed on an NT-MDT Solver Pro AFM (Moscow, Russia) in semi-contact mode, using a rectangular cantilever equipped with an NT-MDT NSG01 crystal silicon gold-coated tip. NP solutions were diluted 1:5 in double-deionized water immediately before analysis. A 50 μL drop of the suspension was deposited onto freshly cleaved inert mica supports, incubated for 10 min at ambient temperature, and then dried under argon gas flow. AFM diameter and height measurements were conducted selecting 20 particles from three different scanning areas (10 × 10 µm). Images were processed with the open-source software Gwyddion ver. 2.62.

#### 2.3.6. Lyoprotection

To protect the NPs during the freeze-drying process, four different concentrations of mannitol and MD-19 were used. Final concentrations of 0.5%, 0.75%, 1%, and 1.25% (*w/v*) were obtained by dissolving the powder in the formulation for 10 min until the colloid was clear. The suspensions were ultimately frozen and kept at a temperature of −80° C until freeze drying began.

#### 2.3.7. Resuspension Test and Milling

Resuspension tests were conducted by dissolving the lyophilized NP (lyoNP) cake in double-deionized water with 0.25% and 0.5% *v/v* of acetic acid at a concentration of 1 mg/mL. The DLS profiles of all samples were then analyzed and compared. The best-performing lyoNP cake was analyzed through DLS. For this purpose, the cake was milled using a Mixer Miller Retsch MM 400 (Retsch GmbH, Haan, Germany) equipped with dedicated 25 mL steel jars and milling steel balls (12 mm ø), with a single milling cycle of 30 Hz for 10 s.

#### 2.3.8. Attenuated Total Reflection—Fourier Transform Infrared Spectroscopy (ATR-FTIR)

The IR spectra of CS, NaTPP, MD-19, empty lyoNPs, and loaded lyoNPs were collected using a Nicolet iS50 Fourier Transform Infrared (FTIR) spectrometer (Thermo Fisher Scientific, Waltham, MA, USA) equipped with a horizontal attenuated total reflectance (ATR) crystal (Diamond). Spectra were recorded in transmittance mode using dried or lyophilized samples placed directly onto the ATR crystal. Each spectrum was the result of the average of 64 scans at 4 cm^−1^ resolution. Measurements were recorded between 4000 and 400 cm^−1^. Images were recorded and analyzed using the dedicated software Jasco FTIR-4700 Spectra Manager 2020.

### 2.4. Encapsulation Efficiency (EE)

The NP formulation was subjected to ultracentrifugation for 1 h at 38.000 rpm using an L-90K ultracentrifuge (Beckman Coulter, Brea, CA, USA) equipped with a SW 40 Ti rotor at 4 °C. The supernatants were then concentrated using a HetoVacuum centrifuge concentrator (Thermo-Fisher, Waltham, MA, USA). The EE% was expressed in terms of (1) total phenolic content (TPC-EE%) by measuring the TPC of the concentrated supernatants through the Folin–Ciocalteu method and (2) OH-Tyr and Tyr content (OH-Tyr + Tyr EE%) by measuring the amount of OH-Tyr and Tyr in the concentrated ultracentrifugation supernatants after hydrolysis. In both cases, the EE% was calculated using the following equation:

(1) $$\mathrm{EE}\;(\%)=\frac{\;\mathrm{Extract}-\mathrm{Supernatant}\;}{\mathrm{Extract}\;}\;\times \;100$$

where “extract” represents the TPC or the concentration of OH-Tyr + Tyr of the OPFE extract at the same dilution used in the colloidal formulation, and “supernatant” is the TPC or the concentration of OH-Tyr + Tyr in the particle-free solution collected after ultracentrifugation.

### 2.5. Antioxidant Capacity

#### 2.5.1. ABTS

OPFE and OPFE NP antioxidant capacity (AOC) was evaluated through an ABTS radical-scavenging assay following the method proposed by Fierri et al. [23], with a few modifications. Briefly, the ABTS working solution was produced by mixing a 7 mM ABTS solution with a 2.6 mM potassium persulfate solution in equal quantities and allowing the mixture to react for 12 h in the dark. The radical solution was then diluted with 10 mM sodium acetate buffer, pH 4.0, to reach an absorbance of 0.75 at 734 nm immediately before the analysis. The assay was performed on a 96-well microplate (Sarstedt, Nümbrecht, Germany) by mixing 20 μL of standard extract or NPs with 200 μL of ABTS and subsequently incubating the plate in the dark. Absorbance decreases at 734 nm were measured every 5 min for a total of 35 min. The results were expressed as Trolox-equivalent antioxidant capacity (TEAC, mM) using a Trolox calibration curve from 0.5 mM to 0.015 mM.

#### 2.5.2. Ferric-Reducing Antioxidant Power (FRAP)

The FRAP assay was conducted using 96-well microplates as described by Fierri et al. [23], with a few adaptations. Briefly, 20 μL of standards, OPFE, or NPs was mixed with 280 μL of freshly prepared FRAP reagent. The plate was incubated at 37 °C for 30 min, and the absorbance was read at 593 nm. The FRAP reagent was prepared by mixing 10 volumes of 300 mM acetate buffer at pH 4.0, 1 volume of 2,4,6-tripyridyl-s-triazine (TPTZ) 10 mM, and 1 volume of 20 mM ferric chloride solution in 40 mM HCl. Similarly to the ABTS assay, measurements were taken every 5 min in a time window of 35 min. Results were expressed as Trolox-equivalent antioxidant capacity (TEAC, mM) using a Trolox calibration curve from 1 mM to 0.015 mM.

#### 2.5.3. Fenton Reaction

The Fenton reaction was performed as previously described with a few modifications [24]. In brief, ammonium iron (II) sulfate was used as a reference standard for the reaction titration at a concentration of 3.55 µM. Before use, ammonium iron (II) sulfate was oxidized in air and stored in small aliquots at −80 °C. Different preparations containing ammonium iron (II) sulfate were mixed with increasing concentrations of powdered NPs (from 250 to 1000 µg/mL), and after the addition of AA (20 µM) and the fluorescent dye dihydrorhodamine123, all samples were monitored every 3 min for 90 min. Each sample was analyzed in triplicate using a fluorescence plate reader (Fluoroskan Ascent, Thermo Electron Corporation, Vantaa, Finland; excitation 485 nm; emission 520 nm).

### 2.6. Cell Assays

#### 2.6.1. Cell Culture

The macrophage-like THP-1 cell line (AddexBio, C0003024) was cultured as previously described [25]. The choice of such a cell line was guided by previous evidence of its high reliability in evaluating oxidative stress and antioxidant signaling pathways [26,27]. Endotoxin contamination during cell culture was routinely inhibited with the chromogenic Limulus Amebocyte Lysate assay (Thermo Fisher Scientific).

#### 2.6.2. Cell Viability Assay

Early apoptosis and cell viability were determined using the PE Annexin V-FITC Kit (Bender MedSystems GmbH, Vienna, Austria) and 7-amino-actinomycin D (BD Biosciences, Buccinasco, Italy). PE Annexin V is used to quantitatively determine the percentage of cells within a population that are actively undergoing apoptosis. 7-Amino-actinomycin is a standard flow cytometric viability probe used to distinguish viable from nonviable cells. The THP-1 cell line was cultured at a concentration of 1 × 10^6^ cells/mL in a 24-well plate; then, the medium was replaced, and increasing concentrations (from 250 µg/mL to 1000 µg/mL) of NPs were added. After 1 h, the fluorescence intensities of cells per sample were determined by flow cytometry using the FACS BD Canto cytofluorometer. A minimum of 10,000 cells were collected for analysis by flow cytometry, and quantitative analysis was performed in Image J (NIH, Bethesda, MD, USA). All the assays were performed in triplicate.

#### 2.6.3. Intracellular Reactive Oxygen Species (ROS) Measurement

Intracellular ROS formation was determined using the CellROX Deep Red Flow Cytometry Assay Kit (Thermo Fisher Scientific, Waltham, MA, USA). Briefly, the cell-permeable CellROX Deep Red reagent is essentially non-fluorescent while it is in a reduced state but exhibits a robust fluorogenic signal upon oxidation, providing a reliable measure of ROS in live cells [28]. To explore the effect of NPs on counteracting oxidative stress, increasing concentrations (from 250 to 1000 µg/mL) of lyophilized NPs were added to TPH-1 cells for 1 h before the addition of a 100 μM solution of TBHP for 45 min at 37 °C. Subsequently, CellROX Deep Red reagent at a final concentration of 1000 nM was added to the cells for 45 min at 37 °C and then immediately analyzed by flow cytometry.

### 2.7. Statistical Analysis

Statistical analyses were performed using GraphPad Prism version 8.0.0 (GraphPad Software, San Diego, CA, USA). The data were expressed as mean ± standard error of the mean (SE) or standard deviation (SD). Differences between the experimental groups were assessed using one-way analysis of variance (ANOVA) followed by the post-hoc Tukey test.

## 3. Results and Discussion

### 3.1. OP and LOP Characterization

It is known that humidity, together with microbial and insect proliferation, has a strong impact on the total phenolic content of OP, causing a substantial reduction in secoiridoid and phenylpropanoid concentrations [29]. Cecchi and coworkers assessed the total phenolic content and sensory properties of pitted OP by comparing several drying methods and concluded that oven drying has a negative impact on the concentration of phenolic compounds in OP samples compared to industrial oven drying and freeze drying [29]. For this reason, we opted to freeze dry all batches shortly after collection.

The moisture content of the fresh OP was 63.93% ± 0.01. This value is consistent with the literature, which indicates a percentage close to 70% for two-phase olive pomaces [30]. The TPC was 25.44 ± 0.56 mg GAEq/g of OP dw, a value comparable to that found by Ribeiro et al. [31] but different from that reported in other studies [32,33]. Discrepancies in the literature may be due to the different analytical and extraction procedures but may also be related to differences due to cultivars, seasoning, ripeness degree, and other factors [34,35].

**Figure 1:**
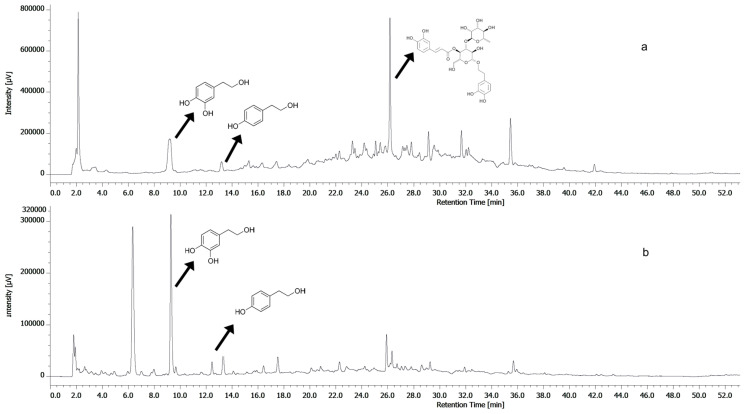
HPLC-DAD chromatogram of non-hydrolyzed (**a**) and hydrolyzed (**b**) OPFE (280 nm). Inset: chemical structure of OH-Tyr (tR A: 9.2 min, tR B: 9.7 min), Tyr (tR A: 13.1 min, tR B: 12.9 min), and verbascoside (tR A: 26.1 min).

Figure 1 shows the hydrolyzed and non-hydrolyzed HPLC profiles of OPFE at 280 nm. Owing to the presence of several secoiridoidic molecules, which are precursors of OH-Tyr and Tyr units, we applied acidic hydrolysis to break the more complex molecules into their simpler constituents [29].

Since the health-promoting effects of olive phenols are mainly ascribed to OH-Tyr and Tyr, we decided to focus on the quantification of these two phenylethanoids. The concentration of OH-Tyr and Tyr turned out to be 4.34 ± 0.25 and 1.33 ± 0.23 mg/g of OP dw, respectively. These values are lower than those found by Bellumori and coworkers [19], i.e., 13.82 ± 0.66 and 1.52 ± 0.05 mg/g dw for OH-Tyr and Tyr, respectively, but in agreement with the TPC values, indicating that the present OP was poorer in phenolic compounds.

### 3.2. NP Production and Characterization

OPFE was used to prepare two different NP formulations at different TPC values (0.08 and 0.12 mg/L of final volume) to evaluate possible dose-dependent effects. An empty version, thus not containing the extract, was also produced. Sizes, polydispersity indexes (PDIs), and ζ-potentials were measured through DLS and NTA, which are appropriate techniques to describe particle dimensions and relative distributions in a liquid formulation, and AFM, by which it is possible to evaluate the average diameter of dried NPs. All measurements are shown in Table 1.

**Figure 2:**
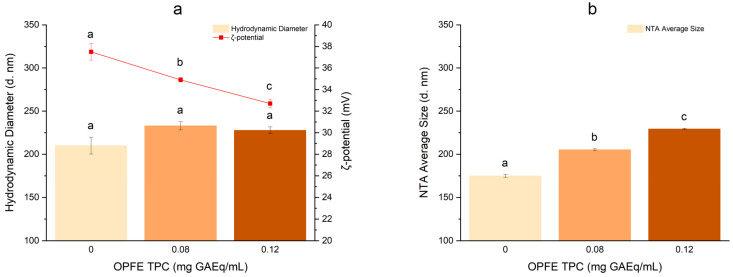
Effect of OPFE concentration on the DLS hydrodynamic diameter of the main peak and ζ-potential of NPs (**a**) and on the NTA average size (**b**). Different lowercase letters indicate significant differences (*p* ≤ 0.05). Color variations refer to increasing OPFE concentrations (GAEq/mL) used for NPs preparation.

**Figure 3:**
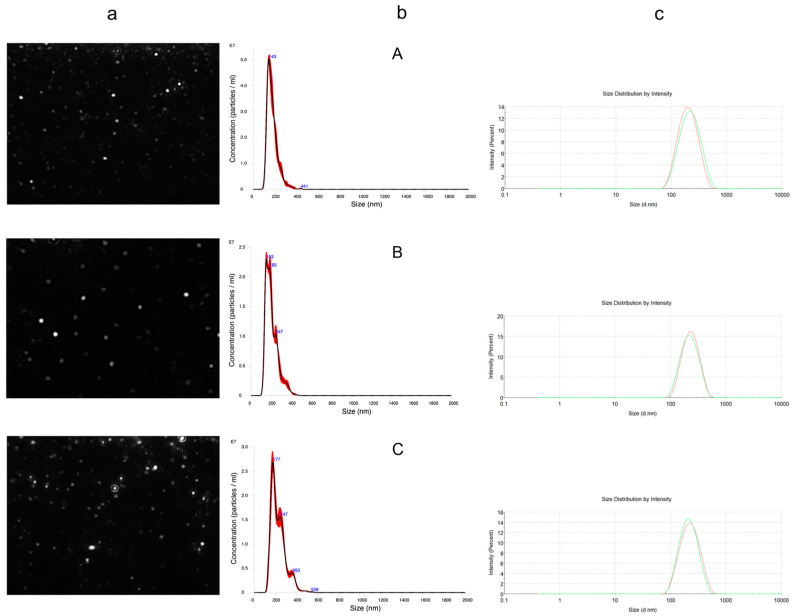
NTA video frame (row a), finite track length adjustment (FTLA) concentration/size graph of particles (row b), and DLS intensity distributions (row c) of empty NPs (**A**), 0.08 mg GAEq/mL NPs (**B**), and 0.12 mg GAEq/mL NPs (**C**). Different colors in row c refer to different experimental replicates.

**Figure 4:**
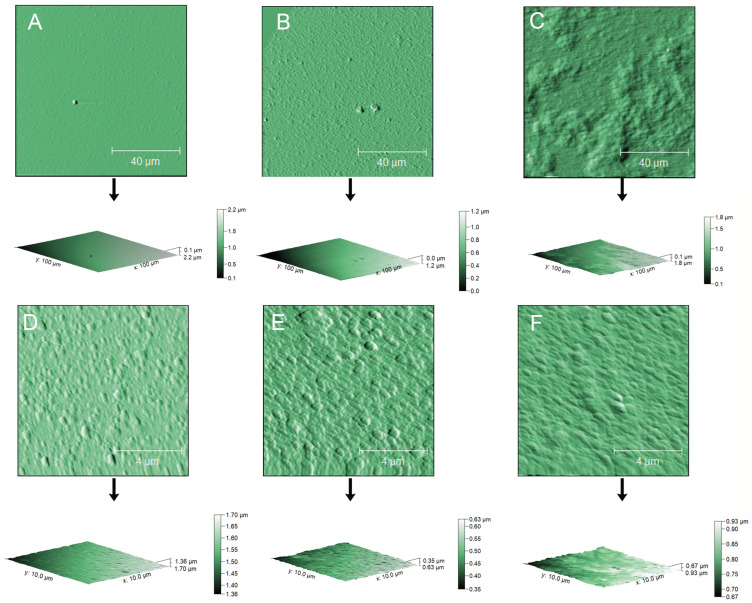
Semi-contact AFM analysis of empty NPs (**A**,**D**) and NPs containing 0.08 mg GAEq/mL (**B**,**E**) or 0.12 mg GAEq/mL (**C**,**F**). The dimensions of the scanning areas were 100 × 100 µm for panels **A**–**C** and 10 × 10 µm for panels **D**–**F**. Samples were diluted 1:5 using double-deionized water and allowed to settle on muscovite micas for at least 10 min before drying through argon flow immediately before scanning.

All formulations showed dimensions in the nanometer range and PDI values below 0.25. The increase in TPC content led to a dose-dependent, statistically significant ζ-potential decrease (empty NPs: 37.50 ± 0.77 mV, 0.12 mg GAEq/mL NPs: 32.77 ± 0.37 mV) and size increase based on NTA analysis (empty NPs: 174.80 ± 1.51 nm, 0.12 mg GAEq/mL NPs: 232.91 ± 4.72 nm), as shown also in Figure 2. However, DLS analysis failed to show differences in particle dimensions as a function of polyphenol concentration. This may be due to the known DLS sensitivity to larger particles, which may lead to a bias toward bigger dimensions. On the contrary, NTA is based on the combination of two different methodologies: light scattering microscopy and charge-coupled device (CCD) camera recording. CCD enables the visualization and recording of the NP flow under the camera lens (Figure 3), allowing for a more accurate size determination [36]. Phenols behave like weak or very weak acids [37], and thus they can be mainly undissociated in the acidic environment provided by the chitosan colloidal dispersion. Considering the strong positive charge of the chitosan deacetylated amino groups, different kinds of interactions may occur between phenols and the chitosan matrix, such as hydrogen bonds and cation–π interactions. The latter are widely present between electron-rich systems like phenol rings and adjacent cations or species containing positive charges [38]. In addition, despite the hydrophilic nature of chitosan, the presence of axial C H bonds in ᴅ-glucosamine leads to the formation of hydrophobic planes both above and below the chitosan layer [39], which might reasonably interact with the more hydrophobic secoiridoids. This complex interplay may justify the increase in size, which might be due to a weaker binding between chitosan and counterions, and the subsequent lowering of ζ-potential due to the masking of the protonated amine groups. This would also justify the incapacity of the formulation to tolerate OPFE concentrations higher than 0.12 mg GAEq/mL, which, in our experiments, caused the spontaneous aggregation of particles. ζ-Potentials lower than −30 mV or greater than +30 mV are indicators of stability against aggregation phenomena, further justifying the reasons behind the collapse of the more concentrated attempts and the good stability of the examples presented in Table 1. The interaction of chitosan with phenolic compounds was also observed in our previously published work [40], where the encapsulation of chlorogenic acid and catechin dramatically altered the overall mechanical properties of chitosan microbeads obtained by dripping into a TPP solution. Different from the present work, in the past study, the presence of phenolic compounds brought a reduction in the bead volume, but this could be due to the different conditions of the process. With respect to AFM measurements, the analysis of the Z-axis of samples returned a height of 12.36 ± 4.37 nm for empty NPs, 29.84 ± 9.35 nm for NPs containing 0.08 mg GAEq/mL NPs, and 30.02 ± 11.70 nm for NPs containing 0.12 mg GAEq/mL (Table 1). The AFM results differ considerably from the DLS and NTA analyses. NPs imaged in semi-contact mode present oriented ovoid macrostructures (Figure 4), which may be the result of the argon flow direction during the drying phase. The analysis of the z-axis presented a very low height in all cases (empty NPs: 12.36 ± 4.37 nm, 0.08 mg GAEq/mL NPs: 29.84 ± 9.35 nm, and 0.12 mg GAEq/mL NPs: 30.02 ± 11.70 nm), which, paired with the large diameter registered, might suggest the collapse of the particles on the x–y axes and/or the occurrence of agglomeration phenomena due to the sudden dehydration coupled with their soft hydrogel structure.

The EE% was evaluated by measuring the amount of non-encapsulated polyphenols in the formulation over the total content of polyphenols in the extract after clearing the supernatant of all particles in suspension. The TPC-EE% of NPs containing 0.12 mg GAEq/mL was 65.21 ± 0.08, while the EE% expressed as the total content of OH-Tyr and Tyr after hydrolysis was 63.96 ± 1.54 (Table 1).

### 3.3. NP Lyoprotection and Resuspension

The dehydration of liquid nanoformulations is of great importance for different reasons, i.e., lower logistic costs, ease of manipulation, and stability of the bioactive compounds. Nevertheless, this process can lead to the agglomeration of NPs with the concomitant loss of their nanoscale dimensions [16]. Wani and coworkers [41] described the successful resuspension of NP cakes by sonicating the pellet for 30 min at 35 kHz in a 1% acetic acid solution, but in our experiment, the procedure was inefficient. In fact, the empty NPs were characterized by higher PDI, indicating the presence of differently sized populations, even after increasing the sonication power and timing using an ultrasonic probe. We encountered similar problems when trying to follow the procedure suggested by Zhao and coworkers [42], in which freeze-dried NPs apparently retained a very monodisperse appearance (PDI < 0.200). In our work, the resuspension of empty NPs was unsuccessful in all cases, regardless of the solvent used. These negative results pushed us to consider an additive to protect NPs during freeze drying. Lyoprotectants are known to prevent particle agglomeration, which can be caused by a multitude of factors and strictly depends on the physiochemical nature of the sample [43]. We assayed the effects of two different lyoprotectants: mannitol and MD-19. Mannitol is a common bulking agent known for its capacity to form a crystalline structure [44], while MD-19 maintains an amorphous structure that reduces the matrix permeability to oxygen, thus helping prevent oxidation [45]. For these experiments, four concentrations of both lyoprotectants were tested on the empty formulation, i.e., 0.50, 0.70, 1.0, and 1.25% *w/v* (final concentration). For comparison, empty NPs were lyophilized without the addition of any lyoprotective agent and used as a control. After lyophilization, cakes were directly resuspended in water or in acetic acid (0.25% *v/v* and 0.50% *v/v*) at a concentration of 1 mg/mL to be comparable with the liquid formulation. In addition to standard DLS outputs like Z-average, PDI, and main peak diameter, we also considered attenuator and correlogram functions of each sample to better assess their quality. All DLS profiles are presented in Table 2. 

NPs containing mannitol performed poorly in the resuspension test in all cases. The main peak diameter and the Z-averages of all samples were similar, with PDIs below 0.300. However, the high attenuator value recorded by the instrument (> 10) suggests that most of the NPs precipitated during sample preparation. Resuspending the cake with 1.25 *w/v* mannitol in 0.50% acetic acid instead returned a more concentrated population of particles (attenuator: 8) with an acceptable PDI (PDI: 0.27 ± 0.12) and a drastic increase in size (main peak: 509.83 ± 28.95 nm), yet the respective correlation function (Figure S1) showed a sample prone to agglomeration and of overall poor quality. These results are somewhat different from those reported by Costa and coworkers [46], but the discrepancy is likely due to the different concentrations of mannitol employed by the authors (10% *w/v*).

Results from the maltodextrin samples were similar to those obtained with mannitol when the cake was resuspended in water and acetic acid at 0.25% *v/v*. The rehydration of the sample containing 0.7% *w/v* of MD-19 using 0.50% *v/v* of acetic acid instead showed an acceptable PDI (0.31 ± 0.01), proving the presence of a main population of NPs with the same attenuator recorded for the original liquid sample (Table 1), and a slight increment in size (main peak: 398.70 ± 24.45 nm) (correlation function: Figure S2). The smooth sigmoid plot of the correlation function showed a single exponential decay linked to the most relevant population in the sample. The discrepancy observed in the Z-average, smaller than the main peak, coupled with the PDI, suggests the presence of a population of particles of very small dimensions, which can be interpreted as being MD-19 particles in the dispersant. From this preliminary screening, we concluded that MD-19, at a final concentration of 0.7% *w/v*, may be effective in protecting empty NPs during lyophilization, but only if powder rehydration is conducted using 0.50% *v/v* acetic acid. This is not surprising since chitosan solubility is driven by its amino groups, which are completely unprotonated at a neutral pH [47], resulting in a very low solubility in water.

To further improve the solubility of the lyophilized NPs, the cake of the sample enriched with MD-19 at 0.7% *w/v* was subjected to a milling step through a laboratory mixer ball mill. The sample was pulverized in a single milling cycle at 30 Hz for 30, 15, and 10 s. The short time intervals selected for this test were strictly related to the rapid increase in temperature inside the grinding jar due to the high energy input provided. Several authors reported how ball milling could be responsible for the changes in the physiochemical characteristics of matrixes and drugs caused by the impact and the temperature produced [48,49]. In the present work, we decided to use the quickest cycle of 10 s to pulverize the lyophilized flakes to prevent any change in the NP structure and any heat-mediated stress. Cycles of 30 and 15 s were too intense, causing sample adhesion on the walls of the jar, probably due to the increase in temperature and mechanical stress. A cycle of 10 s could provide a fine powder, which could be easily removed from the steel container. Powders were ultimately resuspended at a concentration of 1 mg/mL using 0.50% *v/v* acetic acid. The pulverization procedure reduced the polydispersity of both the empty and loaded samples and caused a slight decrease in the main peak size (correlation functions: S3 and S4). Results are presented in Table 3.

### 3.4. ATR-FTIR

**Figure 5:**
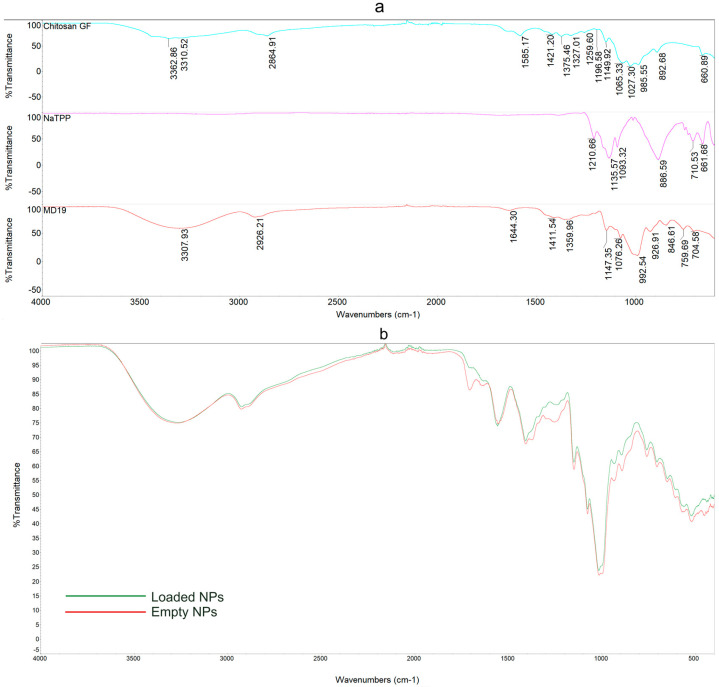
Stacked ATR-FTIR spectra of CS, NaTPP, and MD-19 with peak absorption (**a**) and overlayed ATR-FTIR spectra of loaded (0.12 mg GAEq/mL) and empty NPs (**b**).

Stacked spectra of polymers and overlayed spectra of empty and loaded NPs are shown in Figure 5, while the significant peak absorption regions and their respective assignments are listed in Table 4.

Pure CS presents a broad asymmetric band between 3400 and 3000 cm^−1^, representing the sum of the O-H axial stretching associated with free, intermolecular, and intramolecular bound hydroxyl groups, and two characteristic absorption bands at 3362 cm^−1^ and 3310 cm^−1^, representing the N-H vibration of primary and secondary amines. A peculiar band at 2864 cm^−1^ represents axial C-H stretching. The absorption bands of three types of amides are presented in the region from 1700 cm^−1^ to 1320 cm^−1^, in which we can evaluate the axial deformation of the C=O group linked to the amide I at 1644 cm^−1^, which some authors describe as chitin residues still present in the chitosan powder [50]; the N-H bending belonging to amide II at 1585 cm^−1^; and the C-N stretching of amide III at 1327 cm^−1^. Other absorption bands in the same region are represented by the O-H deformation at 1421 cm^−1^ and the C-H group bending vibration at 1375 cm^−1^. The region at lower frequencies belongs to the C=O and C-O-C symmetric and asymmetric vibration of the polysaccharidic chitosan backbone. The CS spectra are consistent with the literature, aside for minor shifts and differences [41,51,52].

The NaTPP spectrum is characterized by the total absence of bands in the higher wavenumber region and a sequence of strong peaks in the region between 1210 cm^−1^ and 888 cm^−1^, typical of the asymmetrical and symmetrical stretching vibrations of phosphate groups [53,54].

MD-19 manifests a broad and intense band at higher frequencies (3307 cm^−1^), signaling the O-H stretching of H-bonded free alcohols, and a small peak at 2926 cm^−1^, which indicates C-H stretching. The region from 1150 cm^−1^ to 992 cm^−1^ is representative of the C-O-C vibrations of the MD glucose units [55].

Once crosslinked with NaTPP and lyoprotected with MD-19, CS undergoes substantial modifications, and several peaks show rather significative intensity variations and hypsochromic/bathochromic shifts, particularly in the amides and polysaccharidic backbone region. Empty NPs present a broad band at 3269 cm^−1^, larger and less intense than the MD-19 band, suggesting the presence of intermolecular hydrogen bonds, which might be the result of crosslinking. The contribution of CS primary and secondary amine vibrations (at 3362 cm^−1^ and 3310 cm^−1^) is not distinguishable. A new peak at 1704 cm^−1^ is ascribable to the C=O stretching of conjugated aldehyde or acids. In the amide region, empty NPs present a less intense amide I band, with a slight hypsochromic shift (11 cm^−1^) and a more intense and visible shift of the amide II band (28 cm^−1^), which have been reported by other authors during CS/NaTPP interactions [22]. Similarly to de Carvalho et al. [56] and Alehosseini et al. [57], we observed an increase in peak intensity and a bathochromic shift in the angular deformation of CH_2_ (1405 cm^−1^), which may be explained as an increase in the polarization of the carbonyl group upon crosslinking.

The spectral profiles of empty and loaded lyoNPs are, for the most part, superimposable, except for a few differences in peak intensities (3269 cm^−1^ and 1557 cm^−1^), which might be ascribed to the presence of the phenolic extract. Different from the empty counterpart, the peak assigned to the C=O stretching at 1704 cm^−1^ and the band signaling the stretching of the amide III acetyl group (1255 cm^−1^) are missing.

### 3.5. Cell-Free Antioxidant Capacity (AOC)

**Figure 6:**
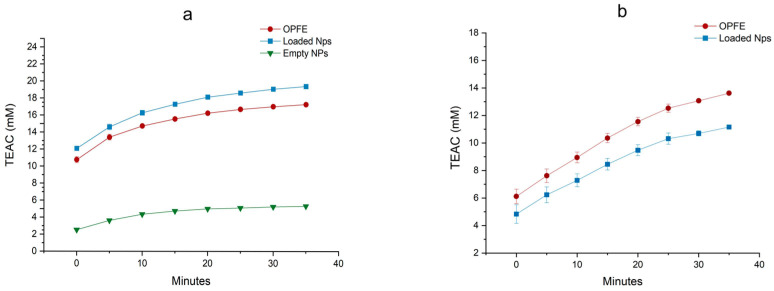
Comparison of the antioxidant capacity measured by ABTS (**a**) and FRAP (**b**) of extract, liqNPs, and empty NPs (ABTS only). Data are expressed as mean ± standard error (SE) from triplicates. Data are expressed as mM Trolox-equivalent antioxidant capacity (TEAC).

Two different spectrophotometric techniques, i.e., ABTS and FRAP, were employed to measure the AOC of OPFE and liqNPs. Results are shown in Figure 6.

**Figure 7:**
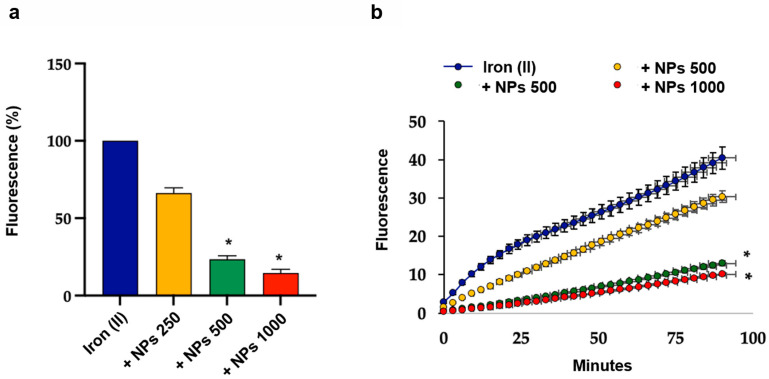
Radical-scavenging effect of the NPs on the ascorbate-driven Fenton reaction. (**a**) Bar graph showing the dose-responsive inhibitory effect of the NPs on the Fenton reaction at 90 min. (**b**) Representative graph showing the fluorescence over time induced by ammonium iron (II) sulfate (iron (II)) and by increasing concentrations of NPs added to iron (II). Data represent the mean ± SD of measurements performed in triplicate in three different experiments: * *p* < 0.001 decrease vs. iron (II).

In the ABTS case, the AOC of the liqNPs is higher than that of the control extract, with a maximum AOC after 35 min of incubation of 17.27 ± 0.12 mM TEAC for the extract, 19.39 ± 0.23 mM TEAC for the loaded liqNPs, and 5.32 ± 0.01 mM TEAC for the empty liqNPs. Extract and loaded liqNPs showed similar kinetic curves. The higher AOC displayed by NPs compared to OPFE is probably caused by the contribution of the carrier itself. The empty liqNPs have an overall faint AOC compared to that of the OPFE and the loaded formulation, but it is enough to dope the signal of the latter. Evaluations from the FRAP assay resulted in an AOC of 13.62 ± 0.09 mM TEAC for the extract and 11.16 ± 0.13 mM TEAC for the loaded liqNPs, while empty liqNPs gave a null result.Results from both assays demonstrate that OPFE phenolics contained in liqNPs can still exert a visible antioxidant effect, even after encapsulation in the CS/TPP matrix. An antioxidant assessment was likewise performed on lyoNPs. Although freeze drying is considered a delicate process, cases of a decline in antioxidant activity or a change in color or properties of food have been mentioned [58,59,60]. To confirm whether freeze drying or the lyoprotectant addition had caused somewhat of a decline in AOC, the lyoNP capacity was assessed through a Fenton reaction assay. This is a sensitive and versatile method that can evaluate the AOC of complex mixtures in biological fluids through the production of hydroxyl radicals (·HO), simulating oxidative stress conditions in cells [61,62]. As shown in Figure 7a,b, lyoNPs were able to significantly reduce the ascorbate-driven Fenton reaction, starting from a concentration of 500 µg/mL. The results agree with those obtained with ABTS and FRAP, confirming the scavenging capacity of the encapsulated polyphenols in the final lyophilized preparation.

### 3.6. Antioxidant Effect of NPs on THP-1 Cells Exposed to Oxidative Stress

**Figure 8:**
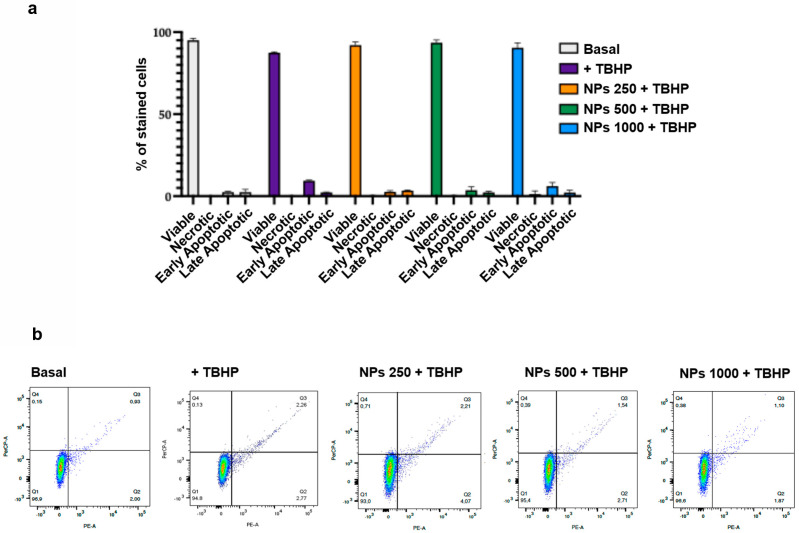
Flow cytometric analysis of the viability of THP-1 cells exposed to oxidative stress and NPs. THP-1 cells were preincubated with increasing concentrations of NPs (from 250 to 1000 µg/mL) before the addition of TBHP (100 µM). (**a**) Bars show the cell viability of control cells, of TBHP, and of NPs (from 250 to 1000 µg/mL). Colors refer to the different samples. Data represent the mean ± SD of measurements performed in triplicate in three different experiments. (**b**) Representative flow cytometry analysis of the effect of control treatment, of TBHP, and of increasing concentrations of NPs on cell viability. Q1: viable cells, Q2: early apoptotic cells, Q4: late apoptotic cells, Q4: necrotic cells.

As lyoNPs displayed significant antioxidant properties in cell-free systems, we ultimately investigated their potential antioxidant effect on THP-1 cells under oxidative stress induced by TBHP. It is well known that the quantification of cell viability serves as the cornerstone for numerous in vitro assays that assess cellular responses to external factors [63,64,65]. Therefore, our initial step involved assessing cell viability using flow cytometry, a highly informative test capable of distinguishing viable, necrotic, and apoptotic cells. Our findings indicate that when THP-1 cells were exposed to oxidative stress with or without preincubation with the lyoNPs, the percentage of live cells remained unchanged compared to control cells. Notably, neither TBHP nor NPs induced early or late apoptosis in THP-1 cells (see Figure 8a,b).

**Figure 9:**
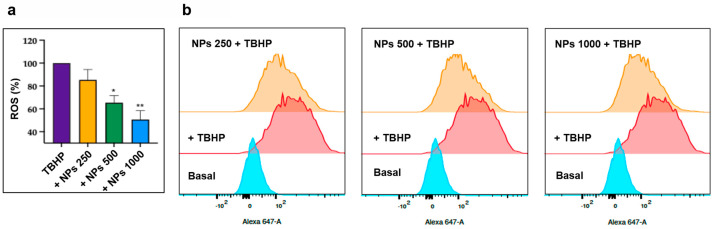
Dose-responsive inhibitory effect of NPs on oxidative stress-induced intracellular reactive oxygen species (ROS) formation. (**a**) Bars show the dose-responsive antioxidant effect of the NPs. THP-1 cells were preincubated with increasing concentrations of NPs (from 250 to 1000 µg/mL) before the addition of TBHP (100 µM). Data represent the mean ± SD of measurements performed in triplicate in three different experiments: * *p* < 0.01 decrease vs. THBP; ** *p* < 0.001 decrease vs. THBP. (**b**) Representative flow cytometry analysis of ROS generation induced by TBHP and the inhibitory effect of the different concentrations of NPs.

Once we confirmed that the lyoNP did not influence cell viability in the 250–1000 µg/mL range, we evaluated whether lyoNPs could inhibit oxidative stress induced by TBHP. Our results show that the preincubation of THP-1 cells with lyoNPs was able to dose-dependently reduce ROS formation, reaching statistical significance at 500 µg/mL (Figure 9).

## 4. Conclusions

In this work, we proposed a sustainable process to convert a liquid, almost monodisperse CS/TPP NP formulation loaded with olive pomace phenolic compounds to a dispersible powder. The gelation process, which valorizes two common by-products, OP and CS, proved itself to be capable of encapsulating phenylethanoids with good efficiency, yielding NPs with dimensions and ζ-potentials that are influenced by the concentration of OP extract. The lyophilization performance of two lyoprotective agents, i.e., mannitol and MD-19, was evaluated using a DLS approach. We showed that a final concentration of 0.7% *w/v* of MD-19 leads to a homogeneous cake, which, once resuspended in 0.5% *v/v* acetic acid, presents a main population of particles of 400 nm in diameter and a PDI of 0.31. It is, therefore, possible to overcome the agglomeration phenomena typically observed when CS is used for NP production. NPs retained their antioxidant capacity not only in cell-free models but also in cultured cells; they thus represent a promising means to counteract oxidative stress using natural molecules derived from the valorization of food by-products.

## Figures and Tables

**Table 1:** DLS measurements, i.e., Z-Average, PDI (polydispersity index), main peak diameter, attenuator, and ζ-potential, NTA average size, AFM average diameter of NPs, and respective total EE% and OH-Tyr + Tyr EE%.

OPFE (TPC) (mg GAEq/mL)	Z-Average (nm)	PDI	Main Peak (nm)	Attenuator	ζ-Potential (mV)	NTA Average Size (nm)	AFM Average Diameter (nm)	TPC-EE%	OH-Tyr + Tyr EE%
0	169.01 ± 5.44	0.19 ± 0.02 a	210.01 ± 9.70 a	7	37.50 ± 0.77 a	174.80 ± 1.51 a	384.34 ± 52.71 a	-	-
0.08	181.02 ± 4.01	0.22 ± 0.01 a	232.91 ± 4.72 a	7	34.91 ± 0.21 b	205.21 ± 1.10 b	451.81 ± 66.57 b	55.68 ± 0.03	47.54 ± 4.77
0.12	180.04 ± 3.95	0.21 ± 0.01 a	227.73 ± 3.92 a	7	32.77 ± 0.37 c	229.20 ± 0.80 c	480.82 ± 67.71 b	65.21 ± 0.08	63.96 ± 1.54

Data are expressed as mean ± standard error (SE) from triplicates. Different lowercase letters within the same column indicate significant differences (*p* ≤ 0.05). OPFE (TPC) concentration is expressed as mg GAEq/mL. -: not applicable.

**Table 2:** Z-Average, polydispersity index (PDI), main peak, and attenuator of empty NPs freeze dried with and without lyoprotectants (mannitol and MD-19).

	Double-Deionized Water				0.25% *v/v* Acetic Acid				0.50% *v/v* Acetic Acid			
	Z-Average (nm)	PDI	Main Peak (nm)	Attenuator	Z-Average (nm)	PDI	Main Peak (nm)	Attenuator	Z-Average (nm)	PDI	Main Peak (nm)	Attenuator
Unprotected	Over	0.85 ± 0.22	82.10 ± 116.11	7	Over	1.00 ± 0.00	215.80 ± 305.19	7	Over	0.54 ± 0.23	414.00 ± 208.74	6
Mannitol protected (% *w/v*)												
0.5	416.00 ± 18.36	0.56 ± 0.47	399.40 ± 59.45	10	342.37 ± 14.33	0.27 ± 0.04	306.27 ± 107.02	10	Over	1.00 ± 0.05	na	8
0.7	498.83 ± 37.46	0.83 ± 0.29	433.27± 83.95	10	278.27 ± 2.15	0.26 ± 0.03	365.77 ± 38.14	10	Over	1.00 ± 0.15	na	7
1	558.50 ± 150.4	0.39 ± 0.28	542.13 ± 213.79	10	307.20 ± 22.68	0.21 ± 0.02	346.90 ± 83.01	10	240.87 ± 16.70	0.44 ± 0.04	421.23 ± 142.23	10
1.25	Over	0.74 ± 0.36	na	9	325.40 ± 18.82	0.36 ± 0.05	497.50 ± 61.40	10	406.00 ± 8.93	0.27 ± 0.12	509.83 ± 28.95	8
MD-19 protected (% *w/v*)												
0.5	Over	0.42 ± 0.19	1315.33 ± 2278.22	9	32.20 ± 0.38	0.98 ± 0.04	496.43 ± 49.45	10	147.30 ± 4.43	0.70 ± 0.05	402.77 ± 32.62	9
0.7	Over	0.24 ± 0.23	683.33 ± 1183.57	8	78.30 ± 8.11	0.98 ± 0.03	666.90 ± 197.26	9	266.23 ± 1.46	0.31 ± 0.01	398.70 ± 24.45	7
1	281.13 ± 42.62	0.75 ± 0.42	518.57 ± 101.27	10	35.44 ± 4.88	0.99 ± 0.02	515.10 ± 268.93	10	169.77 ± 5.11	0.71 ± 0.04	404.70 ± 32.07	8
1.25	73.06 ± 4.29	1.00 ± 10.01	509.27 ± 74.14	11	278.60 ± 42.36	0.31 ± 0.05	236.47 ± 46.33	8	182.40 ± 1.48	0.67 ± 0.01	390.97 ± 20.99	8

Data are expressed as mean ± standard error (SE) from triplicates. The concentration of lyoprotectant in the sample is expressed over the total volume of formulation. na (“not available”) indicates the absence of a reliable measurement, while “Over” indicates a result that is over the limit of detection of the instrument (>1.00 × 10^4^ nm).

**Table 3:** Z-Average, polydispersity index (PDI), mean peak, and attenuator of empty and loaded NPs freeze dried with MD-19 (0.7% *w/v*) and pulverized using a laboratory ball mill (30 Hz, 10 s), resuspended in acetic acid 0.50% *v/v*.

Lyoprotected NPs (Ball-Milled)				
Lyophilized Sample	Z-Average (nm)	PDI	Main Peak (nm)	Attenuator
Empty lyoNPs	246.45 ± 6.12	0.20 ± 0.04	305.32 ± 6.57	7
Loaded lyoNPs	239.50 ± 19.51	0.29 ± 0.02	341.46 ± 34.24	7

Data are expressed as mean ± standard error (SE) from triplicates.

**Table 4:** ATR-FTIR peak regions with their assignment identification.

Compound	Wavenumber (cm^−1^)	Peak Assignments
CS	3400–3000	O-H stretching of H-bonded alcohols, overlapping N-H vibration
	2864	C-H stretching
	1644	C=O stretching
	1585	N-H bending of amide II band
	1421	O-H deformation and bending vibration
	1375 1327 1255	C-H group bending vibration C-N stretching of amide III band Acetyl group, amide III
	1150–892	C-O and C-O-C symmetric and asymmetric vibration of polysaccharidic backbone
NaTPP	1210	P-O stretching
	1130	O-P=O symmetric and asymmetric stretching
	1090	PO_3_ symmetric and asymmetric stretching
	888	P-O-P bridge stretching
MD-19	3600–3000	O-H stretching of H-bonded alcohols
	2926	C-H stretching
	1150–992	C-O and C-O-C symmetric and asymmetric vibration of polysaccharidic backbone
Empty and Loaded NPs	3269–3000	O-H stretching of H-bonded alcohols, intermolecular hydrogen bonding
	2926	C-H stretching
	1704	C=O stretching of conjugated acids/aldehydes
	1633	C=O stretching
	1557	N-H bending and C-N stretching from amide II band
	1405	CH_3_ asymmetric deformation or bending vibration
	1372	C-H groups bending vibration

## Data Availability

Data are available on request from the authors.

## Supplementary Materials

The following supporting information can be downloaded at: https://www.mdpi.com/article/10.3390/antiox13121522/s1. Figure S1—Raw Correlation Data of empty NPs lyoprotected with mannitol (1.25% *w*/*v*) and resuspended in 0.5% *v*/*v* acetic acid. Inset: example of DLS D data output of a single experiment; Figure S2—Raw Correlation Data of empty NPs lyoprotected with MD19 (0.7% *w*/*v*) and resuspended in 0.5% *v/v* acetic acid. Inset: example of DLS data output of a single experiment; Figure S3—Raw Correlation Data of empty NPs lyoprotected with MD19 (0.7% *w*/*v*), subjected to a single milling cycle of 30 Hz for 10 s, and resuspended in 0.5% *v*/*v* acetic acid. Inset: example of DLS data output of a single experiment; Figure S4—Raw Correlation Data of NPs containing 0.12 mg GAEq/mL, lyoprotected with MD19 (0.7% *w*/*v*), subjected to a single milling cycle of 30 Hz for 10 s, and resuspended in 0.5% *v/v* acetic acid. Inset: example of DLS data output of a single experiment.
