# Supplementary material for: Formulation, Characterization, and Antioxidant Properties of Chitosan Nanoparticles Containing Phenolic Compounds from Olive Pomace
Source: Antioxidants (Basel). 2024 Dec 12;13(12):1522. doi: 10.3390/antiox13121522 (PMC11726745; doi:10.3390/antiox13121522)
Supplement: Supplementary file 1 [file antioxidants-13-01522-s001.zip › antioxidants-3343463-supplementary.pdf]

Formulation, characterization, and antioxidant properties of chitosan nanoparticles containing phenolic compounds from olive pomace.

Ilaria Fierri[1], Roberto Chignola[1], Chiara Stranieri[2], Edoardo Giuseppe Di Leo[2], Maria Bellumori[3], Sara Roncoletta[1], Alessandro Romeo[4], Federico Benetti[5], Anna Maria Fratta Pasini[2], Gianni Zoccatelli[1]*

1 Department of Biotechnology, University of Verona, 37134 Verona, Italy

2 Department of Medicine, Section of Internal Medicine D, University of Verona, 37134 Verona, Italy

3 Department of NEUROFARBA, University of Florence, 50019 Sesto Fiorentino, Florence, Italy

4 Department of Computer Science, University of Verona, 37134 Verona, Italy

5 ECSIN-ECAMRICERT SRL Laboratory, 35127 Padua, Italy

* Corresponding Author: Gianni Zoccatelli, PhD

Department of Biotechnology - University of Verona

Strada Le Grazie, 15 - CV1

37134 Verona, Italy

Tel: +39 045 8027952

Fax: +39 045 8027929

e-mail: gianni.zoccatelli@univr.it

Supplementary Information

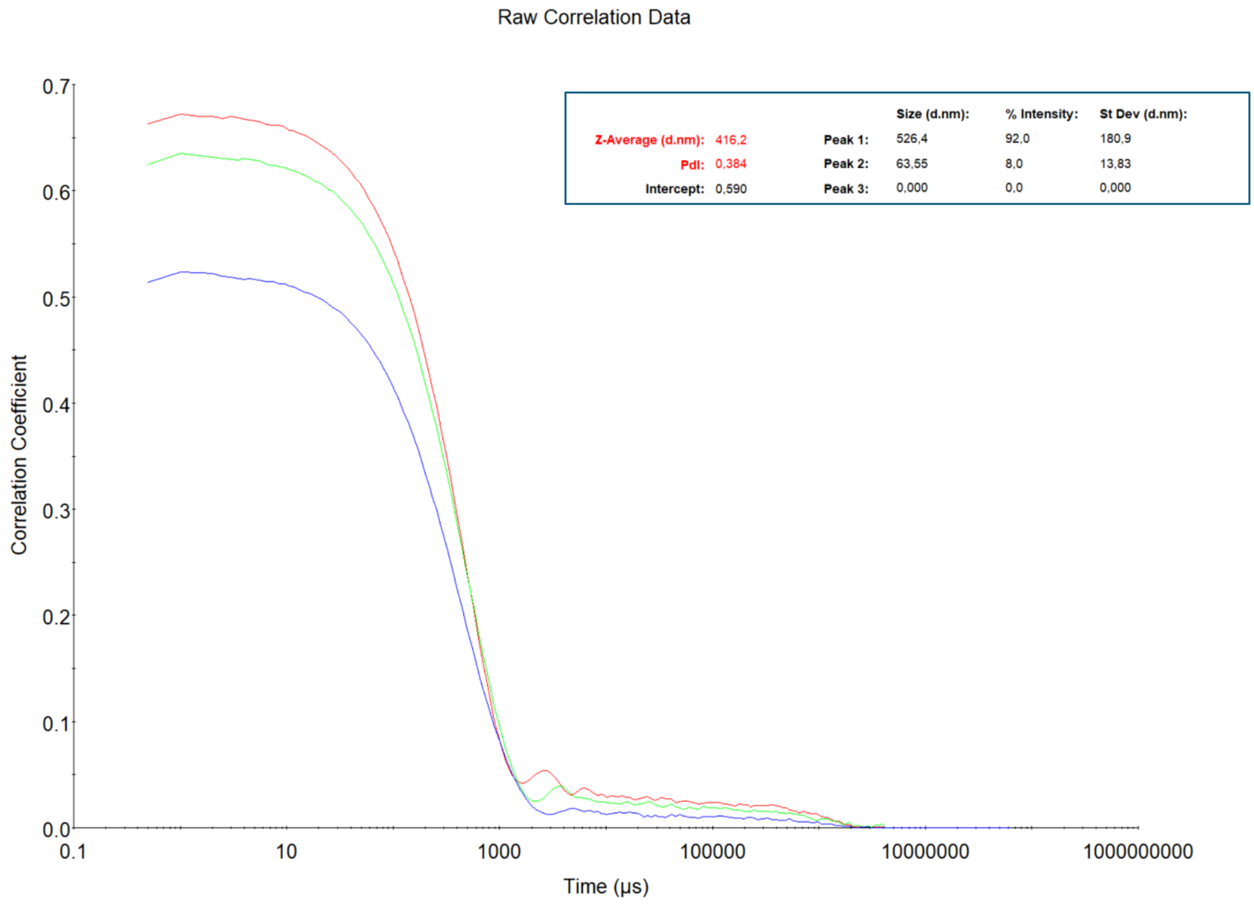

Figure S1 – Raw Correlation Data of empty NPs lyoprotected with mannitol (1.25% w/v) and resuspended in 0.5% v/v acetic acid. Inset: example of DLS D data output of a single experiment.

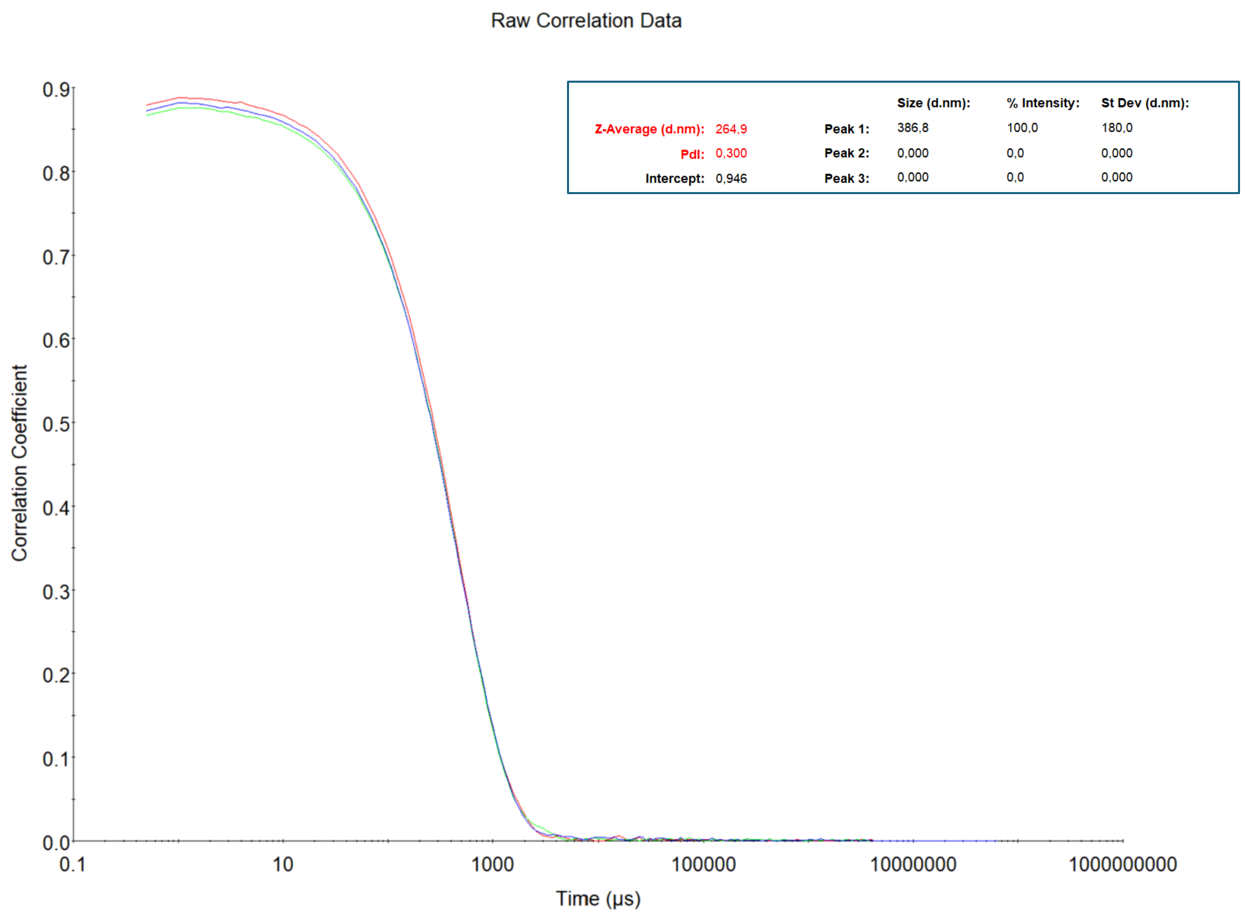

Figure S2 – Raw Correlation Data of empty NPs lyoprotected with MD19 (0.7% w/v) and resuspended in 0.5% v/v acetic acid. Inset: example of DLS data output of a single experiment.

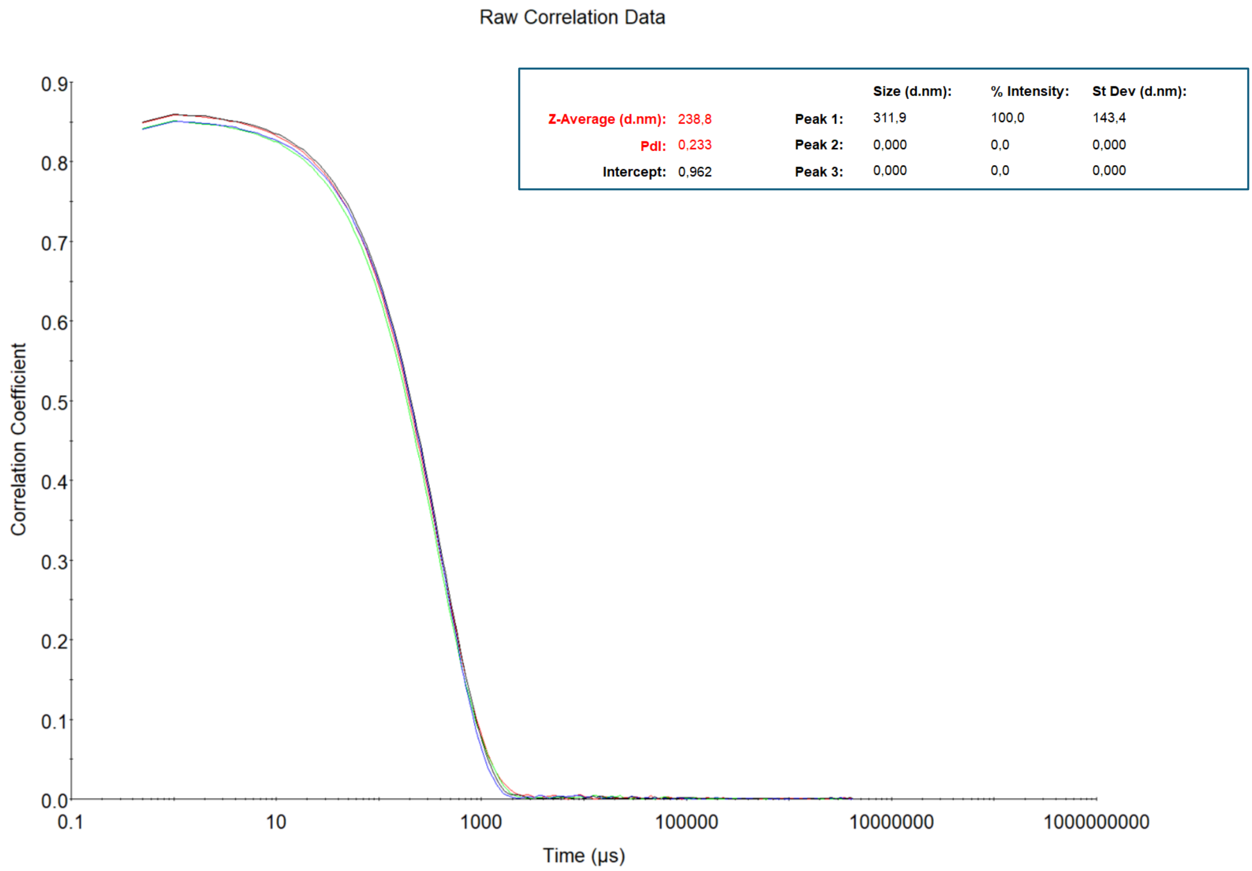

Figure S3 – Raw Correlation Data of empty NPs lyoprotected with MD19 (0.7% w/v), subjected to a single milling cycle of 30 Hz for 10 seconds, and resuspended in 0.5% v/v acetic acid. Inset: example of DLS data output of a single experiment.

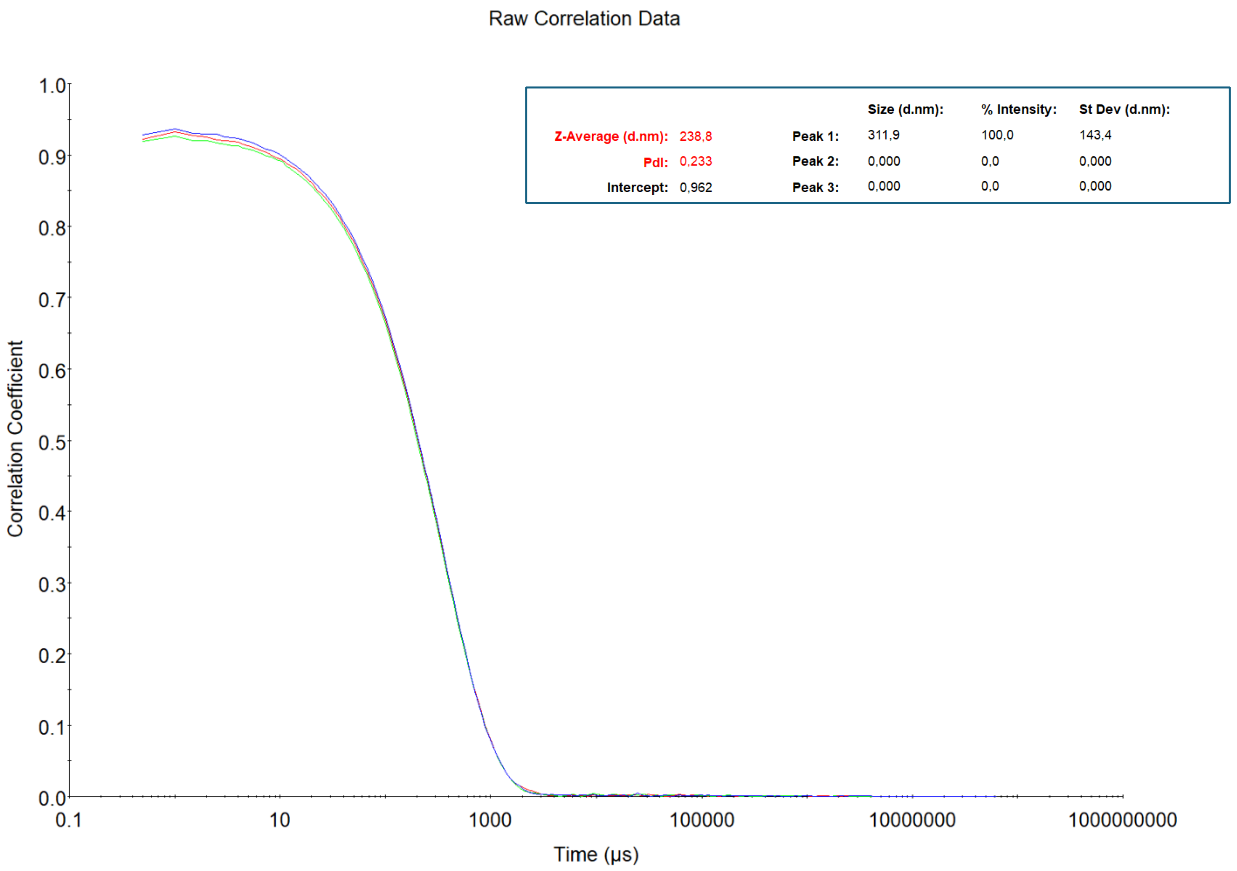

Figure S4 – Raw Correlation Data of NPs containing 0.12 mg GAEq/mL, lyoprotected with MD19 (0.7% w/v), subjected to a single milling cycle of 30 Hz for 10 seconds, and resuspended in 0.5% v/v acetic acid. Inset: example of DLS data output of a single experiment.
